# miR-375 and miR-205 Regulate the Invasion and Migration of Laryngeal Squamous Cell Carcinoma Synergistically via AKT-Mediated EMT

**DOI:** 10.1155/2016/9652789

**Published:** 2016-12-19

**Authors:** Bin Wang, Kexing Lv, Weixiong Chen, Jing Zhao, Jie Luo, Jianhui Wu, Zenghong Li, Hao Qin, Thian-Sze Wong, Weiqiang Yang, Qing-Ling Fu, Wenbin Lei

**Affiliations:** ^1^Department of Otorhinolaryngology, The First Affiliated Hospital of Sun Yat-Sen University, Otorhinolaryngology Institute, Sun Yat-Sen University, Guangzhou, China; ^2^Department of Otorhinolaryngology, The First People's Hospital of Foshan, Foshan, China; ^3^Department of Surgery, Queen Mary Hospital, The University of Hong Kong, Pokfulam, Hong Kong; ^4^Otorhinolaryngology Hospital, The First Affiliated Hospital, Sun Yat-Sen University, 58 Zhongshan Road II, Guangzhou, Guangdong 510080, China

## Abstract

Previous studies have found that miR-375 and miR-205 were significantly dysregulated in laryngeal squamous cell carcinoma, which contributed to the invasion and migration of LSCC. However, the mechanisms of miR-375 and miR-205 regulating the invasion and migration of LSCC remain unknown. qRT-PCR was performed in 40 pairs of tissue samples to investigate the expression of miR-375 and miR-205 in LSCC and paracarcinoma tissues. To investigate whether or not miR-375 and miR-205 regulated the invasion and migration of LSCC synergistically via AKT-mediated epithelial-mesenchymal transition, miR-375 mimic and miR-205 inhibitor were transfected into SNU899 cells and miR-375 inhibitor and miR-205 mimic were transfected into SNU899 cells, respectively, with or without AKT inhibitor. Then the expressions of miR-375 and miR-205 were validated by qRT-PCR, cell migration and invasion were determined by wound healing assay and transwell invasive assay, and western blot analysis was performed to detect the expression of related proteins. Our results showed that miR-375 and miR-205 regulated the invasion and migration of LSCC via AKT-mediated EMT synergistically. In conclusion, our findings provided not only new information about the molecular mechanism of miRNAs regulating invasion and migration of LSCC, but also a theoretical principle for potential targeting therapy of laryngeal squamous carcinoma.

## 1. Introduction

Laryngeal squamous cell carcinoma (LSCC) is the second most common squamous cell carcinoma in the head and neck [1]. LSCC accounted for 1.1% of all new cancers and led to 1.0% of all cancer-related deaths in the world in 2012 [2]. Thanks to the constant improvement in diagnosis and treatment, the five-year relative survival rate of patients with LSCC dramatically decreased in the past 40 years [3]. As the main biology characteristics of cancers, invasion and migration are responsible for more than 90% of cancer-related deaths [4]. Cervical lymph node migration is common in LSCC, especially in supraglottic lesions of which the rate of cervical lymph node migration is as high as 55% [5]. Therefore, studies about the molecular mechanisms of invasion and migration of LSCC are critical for improving the prognosis of patients with LSCC.

Epithelial-mesenchymal transition (EMT) is a process in which epithelial cells with polarity translate into mesenchymal cells with increased motility which are more likely to move freely in the matrix. EMT plays an important role in multiple physiologic and pathophysiologic processes, such as embryogenesis, invasion and migration of tumors, and chemotherapy-resistance of cancers [6]. Recent studies have found that loss of expression of E-cadherin, an adhesive protein of epithelial cells, is significantly correlated with migration and poor prognosis of LSCC [7, 8]. EMT is a complex process that involves multiple signal pathways and regulating factors. Despite the fact that some existing study found that wnt signal pathway played a role in EMT in LSCC [9], there is still a lot to learn about the regulation of EMT in LSCC.

MicroRNAs (miRNAs), 22 nt in length, are endogenous small noncoding RNAs with high fidelity that repress gene expression by binding to the 3′ untranslated region (3′-UTR) of their target mRNAs [10]. It has been demonstrated that the aberrant expression of miRNAs is involved in tumorigenesis and tumor development [11]. By using microarray analysis, our previous study revealed underexpression of miR-375 and overexpression of miR-205 in LSCC [12].

It has been confirmed that miR-375 plays the role of tumor suppressor, as was seen in our previous study, which inhibits the proliferation, invasion, and migration of laryngeal squamous carcinoma cells and promotes their apoptosis via IGF1R-mediated AKT signal pathways [12].

Unlike miR-375, the expression level of miR-205 varies in tumors and remains controversial in LSCC. Tian et al.'s work showed that miR-205 which could inhibit the proliferation of laryngeal squamous carcinoma cells and promote their apoptosis was, however, underexpressed in LSCC [13]. On the contrary, Zhong and Xiong reported overexpression of miR-205 in LSCC, which instead was believed to have promoted the proliferation and invasion of LSCC [14]. Another study also pointed out that the overexpression of miR-205 activated AKT signal pathway through suppressing PTEN expression in endometrial carcinoma [15]. Therefore, the expression and role of miR-205 in LSCC still need to be further studied.

Above all, in this study we examined whether or not miR-375 and miR-205 regulated the invasion and migration of LSCC synergistically via AKT signaling pathways.

## 2. Materials and Methods

### 2.1. Clinical Samples

Forty pairs of clinical samples, each of which included a piece of carcinoma tissue and a piece of paracarcinoma tissue (>1 cm from cancer margin), were obtained from 40 surgical specimens pathologically confirmed with LSCC and surgically resected in Dept. of Otolaryngology Head-Neck Surgery, the First Affiliated Hospital of Sun Yat-Sen University, Guangzhou, China, from March 2013 to April 2015. Details about collection and storing of samples and the inclusion criteria have been described previously [12]. The Human Research Ethics Committee of Sun Yat-Sen University approved the experimental program (the ethical number [2013  23]).

### 2.2. Extraction of Total RNA

Total RNA was extracted from 40 pairs of frozen tissue samples and human LSCC cells SNU46 and SNU899, using TRIzol (Invitrogen, Carlsbad, California, USA) according to the manufacturer's protocol.

### 2.3. Cell Culture and Transfection

Two LSCC cell lines (SNU899 and SNU46) were described previously [12]. miR-375 mimic and inhibitor, miR-205 mimic and inhibitor, and miRNAs negative control (miR NC) were synthesized by RiboBio Co., Ltd. (Guangzhou, Guangdong, China) ( Table 1). Cell transfection was performed using Oligofectamine (Invitrogen, Carlsbad, California, USA) according to the manufacturer's protocol. MK-2206 2HCI (AKT inhibitor) was purchased from Selleck Chemicals (Houston, Texas, USA) and added to culture media at a final concentration of 2 *μ*M.

### 2.4. Quantitative Reverse Transcription PCR

The primers of miR-375, miR-205, and U6 synthesized by FulenGen Co., Ltd. (Guangzhou, Guangdong, China) were as follows: miR-375: (F) 5′-CAGGGTCCGAGGTATT-3′ and (R) 5′-CTGCTTTGTTCGTTCG-3′; miR-205: (F) 5′-UCCUUCAUUCCACCGGAGUCUG-3′ and (R) 5′-CAGACUCCGGUGGAAUGAAGGA-3′. The expressions of miR-375 and miR-205 were quantified by qRT-PCR using SYBR-Green assays on an ABI 7500 real-time PCR system (Applied Biosystems). The results of U6 qRT-PCR gene expression by 2^−ΔΔCt^ method were used as the control.

### 2.5. Wound Healing Assay

Cells were cultured in six-well plates and transfection was performed when cells grew up to 70% density. Twenty-four hours later, 5 wounds were made on the cell monolayer in each well using a 20 *μ*L pipette tip. RPMI-1640 solution was added to the culture after the cells were washed three times with PBS. Images were then taken under inverted microscope at 0 and 24 h after the wounding, so that the mobility of cells was compared among different wells.

### 2.6. Invasive Assay

The capability of cell invasion was determined by transwell invasive assay. 50 *μ*L mixture of Matrigel (BD Biosciences) and RPMI-1640 (1 : 4) was added to the upper side of the polycarbonate transwell filter (Corning). After being dried in 37°C for 4 hours, 2 × 10^4^ cells were seeded and cultured in 200 *μ*L RPMI-1640. 500 *μ*L RPMI-1640 with 10% FBS were added in the lower chamber to attract cells. After being incubated in 5% CO_2_ at 37°C for 48 hours, cells left on the upper chamber were removed with a cotton swab. The filter was fixed with 95% ethanol for 20 min and then stained with 4 g/L crystal violet for 30 min. Cells were photographed in five independent 20x magnification fields under inverted microscope and counted.

### 2.7. Western Blot Analysis

Cells were collected in lysis reagent (Keygen Biotech. Co., Ltd., Nanjing, Jiangsu, China) according to the manual, through which proteins were harvested. Western blot analysis was performed as described previously [16]. The antibodies used included anti-human GAPDH, PTEN, AKT/phospho-AKT (Ser473; Cell Signaling Technology) and anti-human Snail2, E-cadherin, and vimentin (Abcam).

### 2.8. Statistical Analysis

Each experiment was repeated at least three times. All measurement data were expressed as the means ± SD and analyzed using GraphPad Prism 5 software. Wilcoxon signed-ranks test was employed to analyze the differences in miRNA expression between carcinoma and paracarcinoma tissues. The association of the expression of miR-375 and miR-205 with LSCC patients' clinicopathological features was analyzed by Chi-square test. *p* < 0.05 was considered to have statistical significance.

## 3. Results

### 3.1. The Expression of miR-375 and miR-205 in LSCC

**Figure 1:**
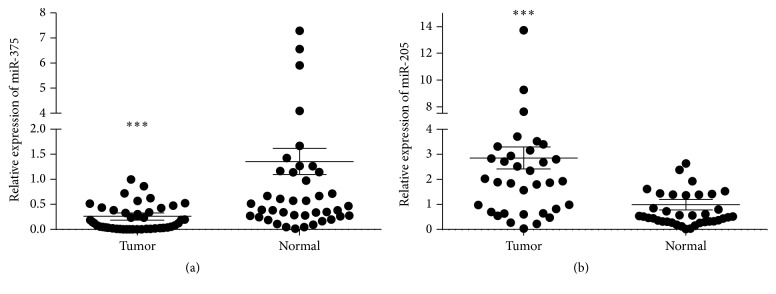
The expression of miR-375 and miR-205 in the 40 pairs of tissue samples. (a) Expression of miR-375 in tumor and normal tissues (qRT-PCR). (b) Expression of miR-205 in tumor and normal tissues (qRT-PCR). *∗∗∗* indicates significant difference revealed when compared with paired normal tissues (*p* < 0.001).

qRT-PCR was conducted in all the 40 pairs of samples to assess the expression levels of miR-375 and miR-205. Significant underexpression of miR-375 (Figure 1(a)) and overexpression of miR-205 (Figure 1(b)) were seen in all the LSCC samples compared with their paired paracarcinoma tissues.

### 3.2. Correlation between Expression of miR-375 and/or miR-205 and Clinicopathologic Features of LSCCs

**Figure 2:**
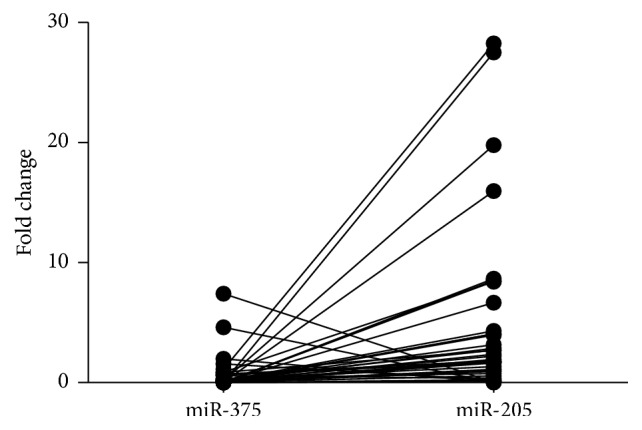
Correlation between the expression of miR-375 and miR-205 in the 40 pairs of tissue samples. Data were analyzed by Spearman correlation test, *r*_*s*_ = −0.577, *p* = 0.004.

As is shown in Table 2, the expression of miR-375 was correlated with pT status (*p* = 0.022) and UICC clinical stage (*p* = 0.013) other than age, nodal status, pathological grade, or anatomic site. The expression of miR-205 was only correlated with anatomic site of LSCCs (*p* = 0.013). The correlation between expression of miR-375 and miR-205 was analyzed by Spearman correlation test (Figure 2), and negative correlation was observed between the expression of miR-375 and miR-205 in 40 pairs of tissue samples.

### 3.3. The Expression of miR-375 and miR-205 in SNU46 and SNU899

**Figure 3:**
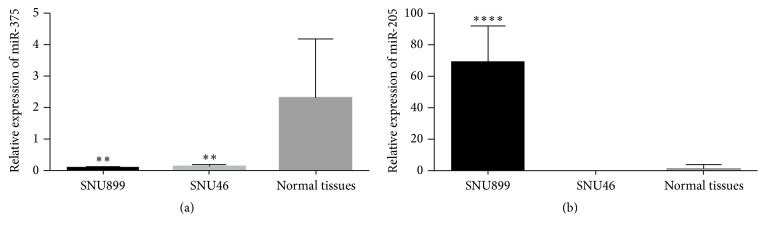
The expressions of miR-375 and miR-205 in SNU46 and SNU899. (a) The expression of miR-375 in SNU46 and SNU899. (b) The expression of miR-205 in SNU 46 and SNU899. *∗* indicates significant difference when compared with paracarcinoma normal tissues of LSCC (*p* < 0.05). *∗∗* refers to *p* < 0.01 and *∗∗∗∗* refers to *p* < 0.001.

We assessed the expression of miR-375 and miR-205 in SNU46 and SNU899 using qRT-PCR. Compared with their expression levels in the 40 paracarcinoma tissue samples, miR-375 expression was lower in both SNU46 and SNU899 (Figure 3(a)) but miR-205 expression was higher in SNU899 and lower in SNU46 (Figure 3(b)). Therefore, we chose SNU899 for further study.

### 3.4. miR-375 and miR-205 Regulated Invasion and Migration of LSCC Synergistically

To investigate whether miR-375 and miR-205 would regulate the invasion and migration of LSCC synergistically, miR-375 mimic, miR-205 inhibitor, and miR NC were cotransfected into SNU899.

**Figure 4:**
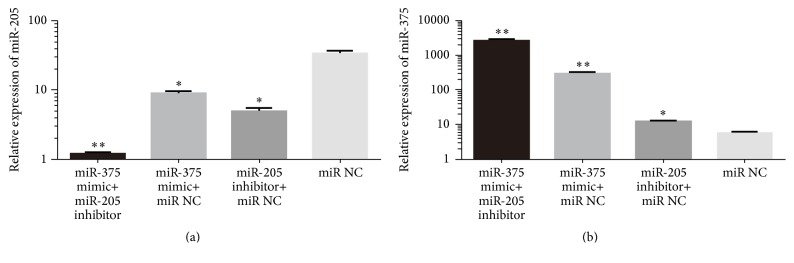
The expression of miR-375 and miR-205 after transfection was conducted in SNU899. (a) The expression of miR-375 in SNU899 after transfection was conducted. (b) The relative expression of miR-205 in SNU899 after transfection was conducted. *∗* indicates significant difference when compared with paracarcinoma normal tissues of LSCC (*p* < 0.05). *∗∗* refers to *p* < 0.01.

The expressions of miR-375 and miR-205 were both detected after transfection using qRT-PCR. As is shown in Figure 4, both miR-375 mimic and miR-205 inhibitor, when transfected alone, could upregulate the expression of miR-375 and downregulate the expression of miR-205 at the same time, which was more significant when miR-375 mimic and miR-205 inhibitor were transfected in combination. This phenomenon hinted to us an interaction between the expressions of miR-375 and miR-205 in LSCC and that the overexpression of miR-375 could inhibit the expression of miR-205, which in return promotes the overexpression of the miR-375.

**Figure 5:**
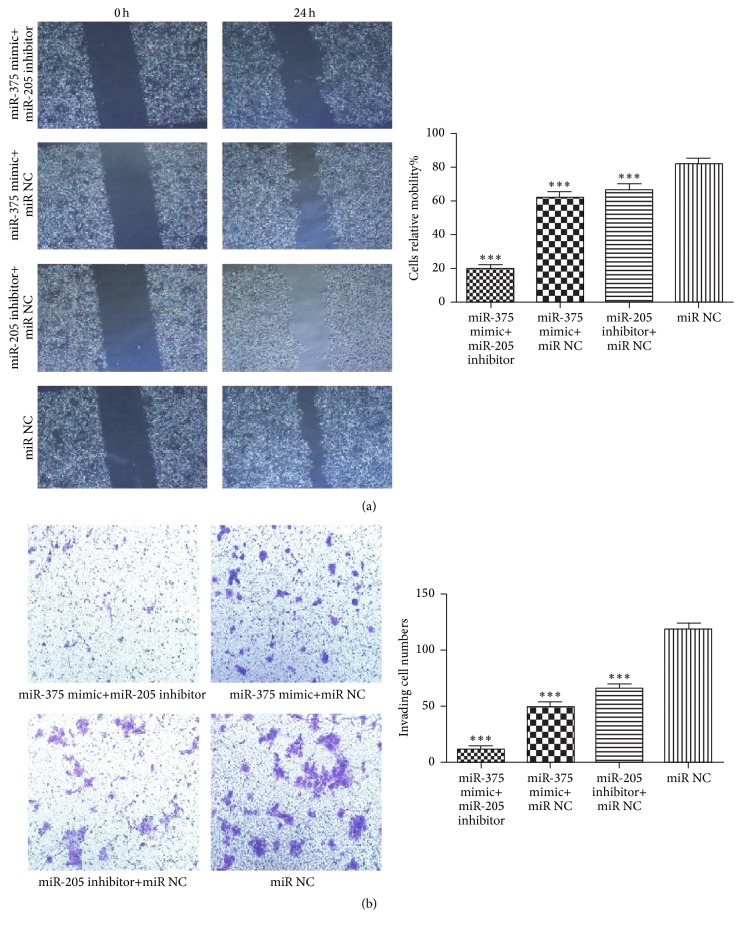
Wound healing assay and transwell invasion assay after transfection were conducted in SNU899. (a) Wound healing assay. (b) Transwell invasion assay. *∗∗∗* indicates significant difference when compared with negative control (*p* < 0.001).

Wound healing and transwell invasion assays were conducted to investigate the migration and invasion of SNU899. The ability of migration and invasion of SNU899 could be suppressed by either miR-375 mimic or miR-205 inhibitor alone and further suppressed by the combined use of both of them (Figure 5). These results suggested that miR-375 and miR-205 regulated the invasion and migration of LSCC cell not separately but synergistically.

### 3.5. Expression of Downstream Proteins of miR-375 and miR-205

**Figure 6:**
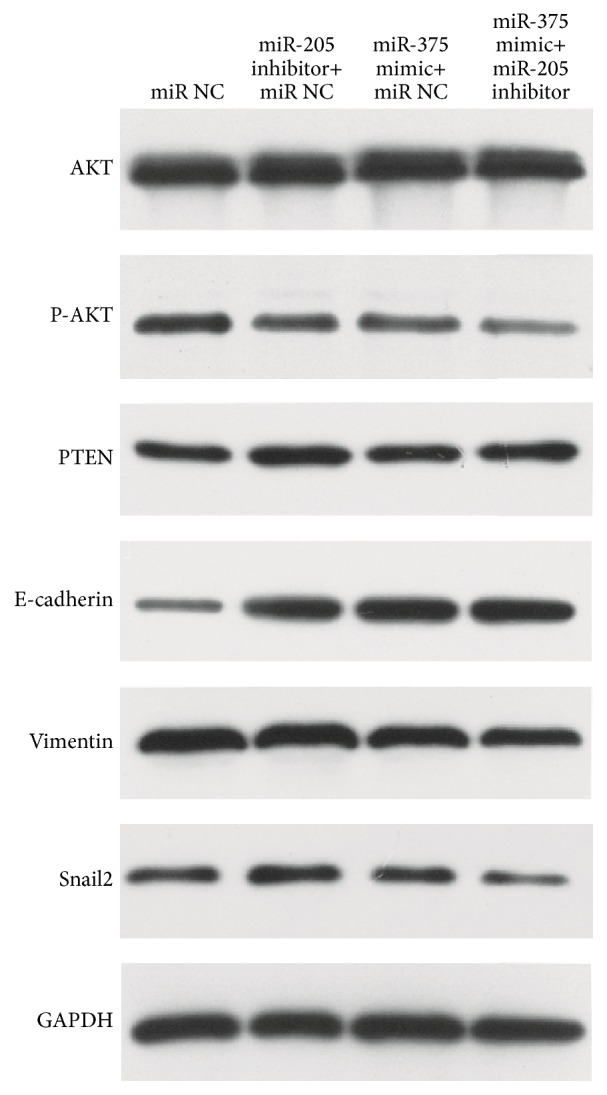
Western blot assay: western blot analysis revealed the effect of miR-205 and miR-375 on the expression of AKT, PTEN, E-cadherin, vimentin, and Snail2. SNU899 cells were transfected with miR-375 mimic+miR-205 inhibitor (miR-375+miR-205 group) or miR-375 mimic+miR negative control (miR-375+NC group) or miR-205 inhibitor+miR negative control (miR-205+NC group) or miR negative control (NC group).

Western blot was conducted to investigate the expressions of PTEN, AKT, p-AKT, Snail2, E-cadherin, and vimentin at the protein level normalized by GAPDH after miR-375 mimic, miR-205 inhibitor, and miR NC were transfected into SNU899. miR-375 mimic or miR-205 inhibitor alone increased the expression of p-AKT and E-cadherin and decreased the expression of Snail2 and vimentin. Such an effect was significantly enhanced when miR-375 mimic and miR-205 inhibitor were cotransfected into SNU899 (Figure 6). And only when miR-205 inhibitor was transfected was the expression of PTEN upregulated (Figure 6). The results indicated that, in LSCC cells, p-AKT, Snail2, E-cadherin, and vimentin were the downstream proteins of miR-375 and miR-205 and their expressions were modulated synergistically by miR-375 and miR-205. However, PTEN was regulated only by miR-205.

### 3.6. AKT Inhibitor Reversed Invasion and Migration of SNU899 Regulated by miR-375 and miR-205

To investigate whether or not miR-375 and miR-205 regulated the invasion and migration of LSCC cell via AKT-mediated EMT, AKT inhibitor was cotransfected with miR-375 inhibitor or miR-205 mimic.

**Figure 7:**
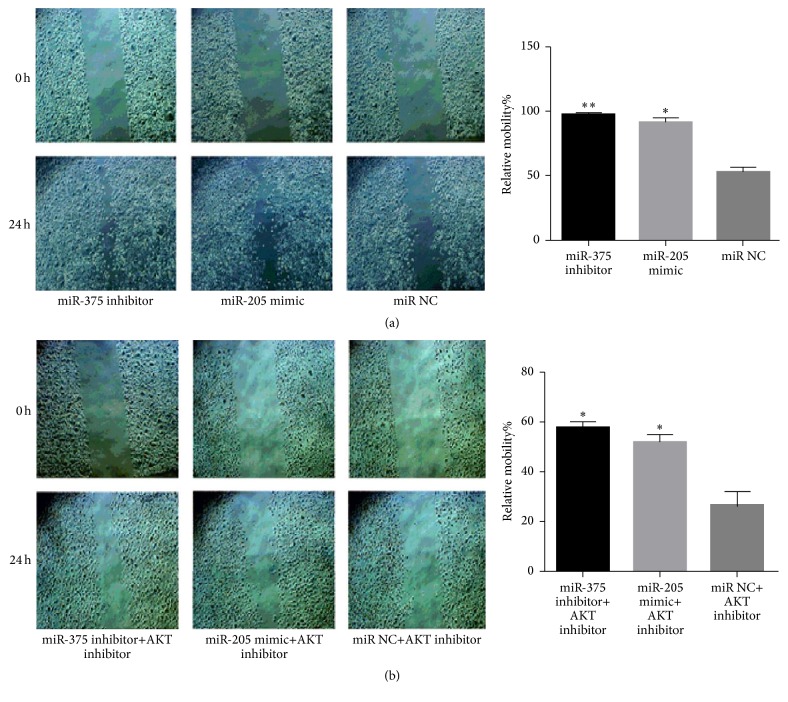
Wound healing assay after transfection with miR-375 inhibitor or miR-205 mimic or miR NC with/without AKT inhibitor. (a) Representative images were taken at 0 and 24 h after scratching without AKT inhibitor. (b) Representative images were taken at 0 and 24 h after scratching with AKT inhibitor. *∗* indicates significant difference when compared with NC (*p* < 0.05). *∗∗* refers to *p* < 0.01.

**Figure 8:**
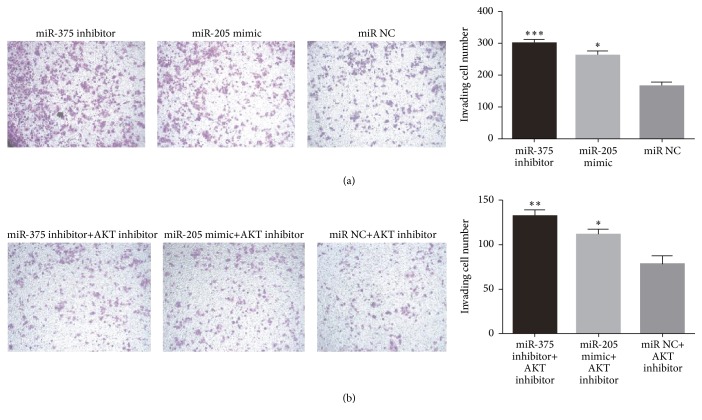
Transwell invasion assay was performed after miR-375 inhibitor and miR-205 mimic were transfected into SNU899 without (a) or with (b) AKT inhibitor. *∗* indicates significant difference (*p* < 0.05) compared with miR NC. *∗∗* refers to *p* < 0.01 and *∗∗∗* refers to *p* < 0.005.

**Figure 9:**
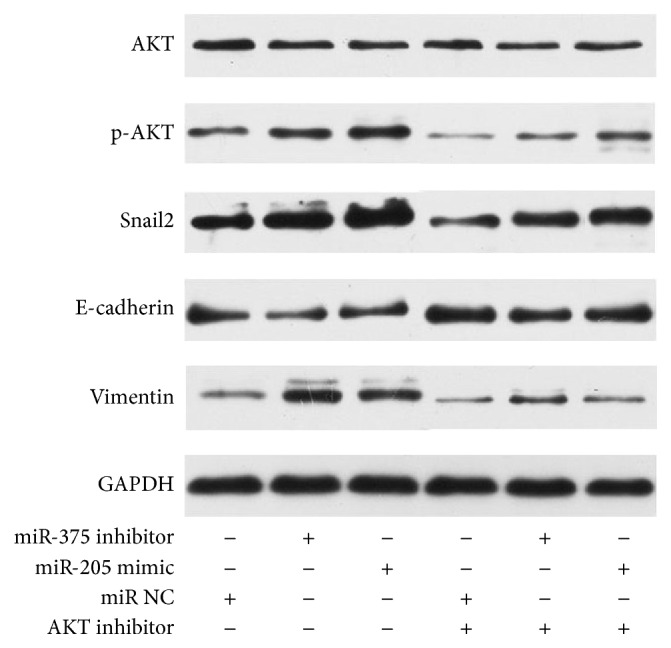
Western blot assay: western blot analysis revealed the effect of miR-205 and miR-375 on the expression of AKT, E-cadherin, vimentin, and Snail2. SNU899 cells were transfected with miR-375 inhibitor or miR-205 mimic or miR negative control with or without AKT inhibitor.

As is illustrated in Figure 7, the promoting effect of miR-375 inhibitor and miR-205 mimic on the invasion of SNU899 was suppressed with the cotransfection of AKT inhibitor, implying that miR-375 and miR-205 regulated the migration of LSCC via AKT signal pathway, which was also suggested by the same results obtained in transwell invasive assay (Figure 8). Further, western blot was performed to investigate the expressions of AKT, p-AKT, Snail2, E-cadherin, and vimentin at the protein level normalized by GAPDH, and the result indicated the effect of miR-375 inhibitor and miR-205 mimic on the expressions of Snail2, E-cadherin, and vimentin of SNU899 was reversed by AKT inhibitor (Figure 9). These demonstrate that miR-375 and miR-205 regulated the invasion and migration of LSCC cell via AKT-mediated EMT.

## 4. Discussion

MicroRNAs have been demonstrated to play critical roles in cell progressions of cancers in recent years [17, 18], but the expression and mechanism of some of them in LSCC remain unclear.

Our previous study [12] found that the expressions of miR-375 and miR-205 were significantly dysregulated in LSCC. In the present study, with the help of qRT-PCR, underexpression of miR-375 and overexpression of miR-205 were demonstrated in LSCC tissue compared with the paracarcinoma normal tissue. miR-375 expression has been found to be significantly downregulated in multiple types of cancers and believed to act as a cancer suppressor [19]. The expression levels of miR-375 and miR-205 vary in different types of cancers. For example, miR-205 is downregulated in prostate cancer [20, 21], breast cancer [22], gastric cancer [23], melanoma [24], and so forth but upregulated in endometrial cancer [25], cervical cancer [26], esophageal squamous cell carcinoma [27], and so forth. The expression of miR-205 in LSCC also varies [20, 21]. The present study found that miR-205 expression was correlated with anatomic site of LSCC, where higher miR-205 expression was seen more frequently in supraglottic LSCC. However, according to Ku et al.'s introduction, SNU-46 was cultured from supraglottic laryngeal carcinoma and SNU899 was cultured from glottic laryngeal carcinoma [28], and in the present study we found that the expression of miR-205 was low in SNU46 but high in SNU899, which was not consistent with the observation in the 40 patient tissue samples. It means that we need more patient tissue samples to analyze the expression of miR-205 in LSCC.

Although small in number, miRNAs were found to control a large set of pathophysiological processes by the fact that multiple miRNAs often work interactively to control individual genes [29]. However, a large number of researches have so far focused on the role of individual miRNAs in different cancers, instead of the interaction among miRNAs. Schouten et al.'s work showed that miR-124 and miR-137 cooperatively controlled caspase-3 activity in hippocampal neural stem cells [30]. Tsukerman et al. found that miR-520-d-5p reduced miR-10b expression by inhibiting the expression of twist in Hela cells [31]. In the present study, we did not only find that miR-375 and miR-205 regulated AKT signal pathway and EMT cooperatively, but also find that there was an interaction between miR-375 and miR-205 in which miR-375 and miR-205 worked interactively to control the expression of each other. According to these results, we hypothesized that the interaction between miR-375 and miR-375 is important for maintaining the ability of invasion and migration of LSCC and that reversing the expression of either one of them could not suppress the invasion and migration of LSCC effectively. Unfortunately, the statistical outcome of our clinical data failed to support this hypothesis. Besides, we did not explore the mechanism by which miR-375 and miR-205 interact with the expression of each other in the present study.

The role of EMT in invasion and migration of cancers has been confirmed, but lots of mechanisms of EMT regulation in LSCC still remain unknown. In the present study, we found that miR-375 and miR-205 regulated EMT process synergistically by regulating the expression of E-cadherin, vimentin, and Snail2 through AKT signal pathways in LSCC. Previous studies have found that IGF1R, as a target gene of miR-375 in LSCC [12], regulates the cell progression of cancers through AKT pathways [32]. PTEN has been found to be a target gene of miR-205 in cancers with overexpression of miR-205 [33, 34] and was proved to be a negative regulator of AKT signal pathways [35]. The AKT signal pathways have been identified to be the upstream signal pathways of EMT process in multiple cancers [36, 37]. Therefore, we confirm that miR-375 and miR-205 regulate the invasion and migration of LSCC via AKT-mediated EMT.

In conclusion, miR-375 acts as a suppressor while miR-205 acts as oncogene, which regulate the invasion and migration of LSCC synergistically via AKT-mediated EMT. Our findings provide not only new information about the molecular mechanism of miRNAs regulating invasion and migration of LSCC, but also a theoretical principle for potential targeting therapy of laryngeal squamous carcinoma.

## Figures and Tables

**Table 1:** The sequence of miR-375 mimic and inhibitor, miR-205 mimic and inhibitor, and miRNA negative control.

	Sequence
miR-205 mimic	5′UCCUUCAUUCCACCGGAGUCUG3′
	3′AGGAAGUAAGGUGGCCUCAGAC5′
miR-375 mimic	5′UUUGUUCGUUCGGCUCGCGUGA3′
	3′AAACAAGCAAGCCGAGCGCACU5′
miR-205 inhibitor	5′CAGACUCCGGUGGAAUGAAGGA3′
miR-375 inhibitor	5′UCACGCGAGCCGAACGAACAAA3′
Mimic negative control	5′UUUGUACUACACAAAAGUACUG3′
	3′AAACAUGAUGUGUUUUCAUGAC5′
Inhibitor negative control	5′CAGUACUUUUGUGUAGUACAAA3′

**Table 2:** Correlation between expression of miR-375 or miR-205 and clinicopathologic features of LSCCs.

Characteristics		miR-375 ↑	miR-375 ↓	*p *	miR-205 ↑	miR-205 ↓	*p *
All samples	(40)	20	20		20	20	
Age				0.53			0.20
≤60	(22)	12	10		9	13	
>60	(18)	8	10		11	7	
pT status				0.02			0.33
Tis -T2	(15)	11	4		9	6	
T3 -T4	(25)	9	16		11	14	
pN status				0.33			0.74
*N0 *	(25)	14	11		12	13	
*N* > 0	(15)	6	9		8	7	
Clinical UICC stage				0.01			0.29
Early stage (I-II)	(11)	9	2		7	4	
Advanced stage (III-IV)	(29)	11	18		13	16	
Pathological grade				0.14			1.00
G1	(10)	7	3		5	5	
G2-G3	(30)	13	17		15	15	
Anatomic site				0.08			0.01
Glottis+subglottis	(29)	17	12		11	18	
Supraglottis	(11)	3	8		9	2	

All patients were staged according to the 2002 Union Form International Cancer Control (UICC) staging classification system for laryngeal cancer.
